# Adjustable sling for the treatment of post-prostatectomy urinary incontinence: systematic review and meta-analysis

**DOI:** 10.31744/einstein_journal/2019RW4508

**Published:** 2019-09-16

**Authors:** Laercio Antonio da Silva, Rogério Simonetti, Edina Mariko Koga da Silva

**Affiliations:** 1 Hospital Israelita Albert Einstein, São Paulo, SP, Brazil.; 2 Universidade Federal de São Paulo, São Paulo, SP, Brazil.

**Keywords:** Suburethral slings, Urinary incontinence, Prostatectomy

## Abstract

Urinary incontinence after prostatectomy has a significant negative impact on the quality of life of the patient. The surgical treatment includes several models of male slings, such as adjustable slings. The objective of this study was to evaluate the effectiveness and safety of adjustable sling in the treatment of post-prostatectomy urinary incontinence. This is a systematic review of literature. The following electronic databases were searched until January 2018: PubMed^®^, Embase, CENTRAL and LILACS. The keywords used in the search strategies were: “prostatectomy” [Mesh], “urinary incontinence” [Mesh] and “suburethral slings” [Mesh]. Randomized clinical trials and observational studies, with or without Control Group, and follow-up of more than 12 months were included. Only one randomized study with high risk of bias was included and it concluded the effectiveness equivalence between adjustable and non-adjustable slings. All other studies were cases series with patients of varying levels of incontinence intensity and history of pelvic radiation therapy and previous surgeries. The meta-analysis for 0 pad in 24 hours demonstrated an effectiveness of 53%. For the 0 to 1 pad test in 24 hours, the meta-analysis resulted in an effectiveness of 69%. Risk factors for surgery failure include prior radiation, severity of post-prostatectomy urinary incontinence, and previous surgeries. The meta-analysis of the extrusion rate was 9.8% and the most commonly reported adverse effects were pain and local infection. Evidence of low quality indicates that adjustable slings are effective for treating post-prostatectomy urinary incontinence, with frequency of adverse events similar to the surgical option considered gold standard (the artificial urinary sphincter implant).

## INTRODUCTION

Urinary incontinence is a well-known sequela of prostate surgeries due to benign or malignant diseases, and it is a frequently undesired outcome, with a significant negative impact on the quality of life of patients.^1^ Most patients presenting post-prostatectomy urinary incontinence (PPI) suffer from intrinsic sphincter deficiency, isolated or associated with detrusor dysfunction,^2^ and present stress urinary incontinence, *i.e*., complaint of involuntary loss of urine upon exertion.^3^

The urinary incontinence rates after surgery of a benign prostate disease are similar in various types of surgical treatment, but tend to be slightly higher after open prostatectomy (0 to 8.4%).^4^ The incidence of urinary incontinence after radical prostatectomy is controversial, since the rates of several studies ranged from 0.8 to 87%.^5-11^ This wide variation is probably due to lack of standardized definition of urinary incontinence, progression time after surgery, diagnostic methods, and characteristics of the population studied.

The surgical treatment of PPI should be indicated only 6 to 12 months after prostate surgery. During this period, some conservative therapies, such as pelvic floor muscle training, interventions in lifestyle, and biofeedback must be considered.^12^ The surgical treatment options include injection of substances that cause volume increase and occlude parts of the urethra, male slings, placement of inflatable balloons that partially occlude the urethra, and artificial urinary sphincter implantation.

The artificial urinary sphincter demonstrated favorable long-term results, and so far has been considered the gold standard for PPI. Nonetheless, this option has well-established surgical complication rates, including urethral erosion (6%), infections (5%), mechanical failures (6%), and need for revision (21% within 5 years, and 50% within 10 years.^13^ Therefore, the use of male slings has recently increased, for providing a simpler and less invasive intervention, not requiring mechanical device manipulation by patients.

Several male sling models have been launched in the last decade and, despite the different mechanisms of action, the primary objective is compression of the urethral bulb with adequate tension, maintaining tension to prevent leaks, and balance between detrusor tension and contractility to avoid urinary retention.^13^ The current male sling models available can be divided as transobturator suburethral, and the more recent adjustable retropubic.

The adjustable retropubic slings are generally inserted in a suburethral position, under the bulbospongiosus muscle, making pressure mainly on the bulbar urethra and, at a lesser extent, on the membranous urethra. In the postoperative follow-up, if there is not enough tension to achieve continence, it can be readjusted by a minimally invasive procedure, specific for each model.^14^ The adjustable slings available comprise Argus^®^ (Promedon, Cordoba, Argentina), ArgusT^®^ (Promedon, Cordoba, Argentina), ReMeex^®^ (Neomedic, Barcelona, Spain) and ATOMS^®^ (AMI, Feldkirch, Austria).

A vast literature on these slings has been recently published, and according to the reports, they are effective, even in cases of sever PPI grave, and in previously irradiated patients. However, no specific systematic review has been published for these models so far.

## OBJECTIVE

To evaluate the effectiveness and safety of adjustable male slings in treatment of post-prostatectomy urinary incontinence.

## METHODS

This systematic review and meta-analysis were conducted in accordance with the PRISMA^15^ protocol, and registered at the International Prospective Register of Systematic Reviews (PROSPERO), with identification code CRD42017082431.

### Types of study

This project included controlled or non-controlled randomized clinical trials and observational studies that evaluated surgical implantation of adjustable sling for treatment of PPI. Only studies with more than 12 months of follow-up were selected.

### Type of participants

Studies with adults presenting with PPI for more than 6 months, with no improvement from conservative treatment, were eligible.

### Type of intervention

Included studies were those that evaluated the surgical implantation of the adjustable sling, compared or not to any other type of surgery for PPI treatment.

### Types of outcome measurements

The primary outcome was cure evaluated by the standardized 24-hour pad test.^16^ Secondary outcomes included adverse events and the need for adjustments in follow-up.

### Research for the identification of studies

Investigations were made in databanks up until January 2018: PubMed^®^, EMBASE, Cochrane Central Register of Controlled Trials (CENTRAL), and Latin American and Caribbean Health Sciences Literature (LILACS) (Tables 1 to 4 [Table t2] [Table t3] [Table t4]). A search was made of ongoing studies at Site ClinicalTrials (https://clinicaltrials.gov/). There was no restriction as to language or date of publication. The reference lists of relevant studies were verified for possible locations of studies.

**Table 1:** Search terms used in all databases

*#1 −* (Surgery OR operative therapy OR operative procedures OR invasive procedures OR operations OR peroperative procedures OR perioperative procedures OR preoperative procedures OR Intraoperative procedures OR Operative Surgical Procedure OR Operative Surgical Procedures OR Operative Procedures OR Operative Procedure)
#2 – (post prostatectomy OR post-prostatectomy OR Prostatectomy OR Prostatectomies OR Suprapubic Prostatectomies OR Suprapubic Prostatectomy OR Retropubic Prostatectomies OR Retropubic Prostatectomy)
*#3 −* (Urinary Urge Incontinence OR Urge Incontinence OR Urinary Reflex Incontinence OR Urinary Stress Incontinence)
#4 − (suburethral Slings OR Suburethral Sling OR Transobturator Tape OR Transobturator Tapes OR Transobturator Suburethral Tape OR Trans-Obturator Tape)
#5 #1 AND #2 AND #3 AND #4

**Table 2 t2:** Search strategy at MEDLINE via PubMed®

#1 randomised controlled trial [pt]
#2 controlled clinical trial [pt]
#3 randomized [tiab]
#4 placebo [tiab]
#5 drug therapy [sh]
#6 randomly [tiab]
#7 trial [tiab]
#8 groups [tiab]
#9 #1 OR #2 OR #3 OR #4 OR #5 OR #6 OR #7 OR #8
#10 animals [mh] NOT humans [mh]
#11 #9 NOT # 10

**Table 3 t3:** Search strategy at Latin American and Caribbean Health Sciences Literature (LILACS) via Latin-American and Caribbean Center for Health Sciences Information (BIREME)

(Pt randomized controlled trial OR Pt controlled clinical trial OR Mh randomized controlled trials OR Mh random allocation OR Mh double-blind method OR Mh single-blind method) AND NOT (Ct animal AND NOT (Ct human and Ct animal)) OR (Pt clinical trial OR Ex E05.318.760.535$ OR (Tw clin$ AND (Tw trial$ OR Tw ensa$ OR Tw estud$ OR Tw experim$ OR Tw investiga$)) OR ((Tw singl$ OR Tw simple$ OR Tw doubl$ OR Tw doble$ OR Tw duplo$ OR Tw trebl$ OR Tw trip$) AND (Tw blind$ OR Tw cego$ OR Tw ciego$ OR TW Mask$ or Tw mascar$ )) OR Mhplacebos PR Twplacebo$ OR (Tw random$ OR Twrandon$ OR Tw casual$ OR Tw acaso$ OR Tw a zar OR Tw aleator$) OR Mh research design) AND NOT (Ct human and Ct animal)) OR (Ct comparative study OR Ex E05.337$ OR Mh follow-up studies OR Mh prospective studies OR Tw control$ OR Tw prospectiv$ OR Tw volunt$ OR Tw volunteer$) AND NOT (Ct animal AND NOT (human and Ct animal)))

**Table 4 t4:** Search strategy at EMBASE via Ovid®

01 random$
02 factorial$
03 crossover$
04 cross over$
05 cross-over$
06 placebo$
07 double$ adj blind$
08 singl$ adj blind$
09 assign$
10 allocate$
11 volunteer$
12 cross-over procedure
13 double-blind procedure
14 randomized controlled trial
15 single-blind procedure

### Study selection

Two authors independently evaluated the studies identified by the literature search as to eligibility. In the case of any uncertainty as to the eligibility of studies based on the title and abstract, the complete text was obtained and examined by the two reviewers. In case of disagreement, a third author was consulted until a consensus was reached. All studies considered eligible were fully obtained and analyzed.

### Methodological evaluation of the studies included

Two authors assessed the included studies in an independent manner as to methodology quality. To evaluate the quality of the methodology in randomized clinical trials, the Cochrane^17^ Collaboration tool was used, and for observational studies, the instrument for critical evaluation of the Chan et al., case series type was utilized.^18^

### Synthesis and data analysis

Measurements of absolute and relative frequencies were calculated with confidence intervals of 95% (95%CI). For the results of continuous variables, central tendencies and 95%CI range were calculated. The unit of analysis was based on the individual patient. For meta-analysis of the extracted data, the Open Meta Analyst software was employed*.*^19^

### Evaluation of heterogeneity

In order to quantify the inconsistencies between the summed estimates, the I^2^=[(Q-df)/Q] × 100% test was used, in which “Q” is the χ^2^ statistic and “df” represents its degrees of freedom. This illustrates the percentage of variability in the estimates of the resulting effect of heterogeneity.^20^ The fixed model was used in the absence of substantial heterogeneity (I^2^<50%), and the random model when there was heterogeneity (I^2^≥50%).

## RESULTS

**Figure 1:**
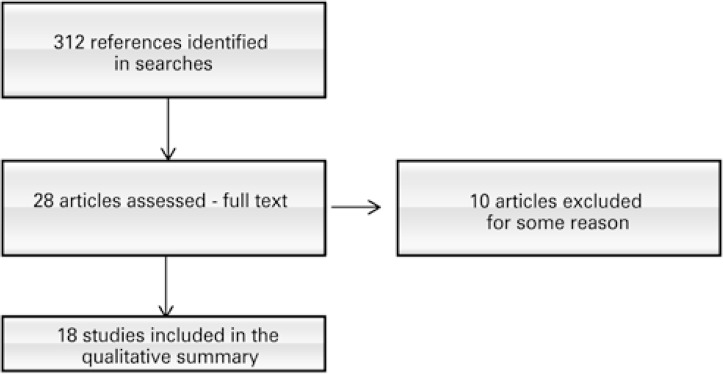
Study flowchart

The search strategy recovered 312 records: PubMed^®^ with 177 references; EMBASE, 113 references; CENTRAL, 11 references; and LILACS, 11 references. Also analyzed were the references of articles relevant to potentially eligible studies and no additional references were located. No ongoing study was located. After the examination of titles and abstracts, eliminating the duplicates of these references, 28 articles were selected for full-text analysis. Ten articles were excluded for not meeting the inclusion criteria, and 18 studies were included in this systematic review (Figure 1).

### Design of the studies included

Only one randomized clinical trial (RCT)^21^ with a limited sample was identified in the search. The other studies included^22-38^were series of prospective and retrospective cases. The characteristics of the studies included are shown on table 5.

**Table 5:** Characteristics of the studies included

Studies	Type of sling	Design	Sample	Severity of PPI	Follow-up period (months)	Definition of cure	Cure rate (%)	Adjustments	Extrusion rate (%)
Lima et al.^(21)^	ArgusT^®^ *versus* Advance^®^	Randomized clinical trial	22	-	18	0-1 pad	ArgusT^®^: 77.8 Advance^®^: 45.5	In 27.3%	-
Angulo et al.^(22)^	ATOM^®^	Prospective	34	Mild: 5.9%	18.5	0-1 pad	85.3	Once	0
				Moderate: 17.6%					
				Severe: 76.5%					
Bauer et al.^(23)^	ArgusT^®^	Prospective	42	Moderate: 7.1 pad/day	28.8	0 pad	61.9	1.7 times	4.8
Bochove-Overgaauw et al.^(24)^	Argus^®^	Retrospective	95	Mild: 13.7%	27	0-1 pad	54	1.5 times	11.5
				Moderate: 48.2%					
				Severe: 43.1%					
Chung et al.^(25)^	Argus^®^	Prospective	25	-	36.2	0-1 pad	92	-	-
Cornel^(26)^	Argus^®^	Prospective	36	-	12	0 pad	50	-	11.1
Friedl et al.^(27)^	ATOMS^®^	Prospective	287	Mild: 11%	31	0-1 pad	64	3.0 times	20
				Moderate: 67%					
				Severe: 22%					
Hoda et al.^(28)^	ATOMS^®^	Prospective	99	Mild: 12%	17.8	0-1 pad	85.5	3.8 times	4
				Moderate: 39%					
				Severe: 49%					
Kim et al.^(29)^	MRS^®^	Prospective	64	Mild: 42.2%	46	0-1 pad	60.9	1.9 times	3.1
				Moderate: 43.8%					
				Severe: 14%					
Leizour et al.^(30)^	Remeex^®^	Prospective	25	Mild: 60%	31	0-1 pad	36	In 60%	16
				Moderate: 20%					
				Severe: 20%					
Lim et al.^(31)^	Argus^®^	Prospective	20	Moderate: 100%	24.7	0-1 pad	85	In 45%	15
Mühlstädt et al.^(32)^	ATOMS^®^	Retrospective	54	Mild: 1.9%	27.5	0 pad	48	4.5 times	9.3
				Moderate: 29.6%					
				Severe: 68.5%					
Navalón-Monllor et al.^(33)^	Remeex^®^	Prospective	24	Severe: 100%	40.7	0-1 pad	100	2.4 times	8
Romano et al.^(34)^	Argus^®^	Prospective	47	-	45	0-1 pad	78.7	In 19.4%	19.1
Romano et al.^(35)^	ArgusT^®^	Prospective	36	Mild e moderate: 22%	45	0 pad	66	In 19.4%	10.4
				Grave: 78%					
Seweryn et al.^(36)^	ATOMS^®^	Prospective	38	Mild: 7.9%	17	0-1 pad	60.5	3.9 times	15.8
				Moderate: 34.2%					
				Severe: 57.9%					
Siracusano et al.^(37)^	ArgusT^®^	Prospective	182	Mild: 11.6%	22	0-1 pad	33	In 30%	9.3
				Moderate: 52.7%					
				Severe: 35.8%					
Sousa-Escandón et al.^(38)^	Remeex^®^	Prospective	51	-	32	0-1 pad	64.7	In 33%	5.9

PPI: post-prostatectomy urinary incontinence.

### Participants

The studies included totaled up 1,170 participants, with ages varying between 46 and 89 years, with an approximate mean age of 70 years. Twelve studies included patients who had received prior radiation therapy, with a proportion of 5% to 44.7%, and eight included patients with prior PPI surgery, with a proportion of 11.8% to 36%. Twelve studies included patients with severe PPI, with a proportion of 7.8% to 76.5%.

### Methodological quality of the studies included

The only RCT^21^presented with low methodological quality, due to uncertain allocation, lack of blinding, and limited sample (Table 6).

**Table 6:** Evaluation of quality of the randomized clinical trial(21)

Domain	Opinion	Description
Appropriate randomization?	Yes	Table with random numbers
Occultation of allocation?	Uncertain	There is no informationo
Blind?	No	Open study
Incomplete outcome data?	No	No report of losses
Free of selective outcome?	Yes	Relevant outcomes reported in results
Free of other biases?	No	No calculation of sample size
		Limited sample
Bias risk	High	

The 17 studies of the case series type presented with moderate to high quality (Table 7).

**Table 7:** Evaluation of quality of the studies included

Study	Clear objective	Appropriate method	Inclusion and exclusion criteria	Recruitment period	Consecutive patients	Appropriate outcomes	Prospective	No significant losses	Final quality
Angulo et al.^(22)^	✓	✓	✓	✓	✓	✓	✓	✓	High
Bauer et al.^(23)^	✓	✓	✓	✓	✓	✓	✓	✓	High
Bochove-Overgaauw et al.^(24)^	✓	✓	✓	✓	✓	✓	✓	✓	High
Chung et al.^(25)^	✓	✓	✓	✓	✓	✓	✓	✓	High
Cornel^(26)^	✓	✓	✓	✓	✓	✓	✓	✓	High
Friedl et al.^(27)^	✓	✓	✓	✓	?	✓	✓	✓	Moderate
Hoda et al.^(28)^	✓	✓	✓	✓	?	✓	✓	✓	High
Kim et al.^(29)^	✓	✓	✓	✓	✓	✓	✓	✓	High
Leizour et al.^(30)^	✓	✓	✓	✓	?	✓	✓	✓	High
Lim et al.^(31)^	✓	✓	✓	✓	?	✓	✓	✓	High
Mühlstädt et al.^(32)^	✓	✓	✓	✓	?	✓	✓	✓	High
Navalón-Monllor et al.^(33)^	✓	✓	✓	✓	?	✓	✓	✓	Moderate
Romano et al.^(34)^	✓	✓	✓	✓	?	✓	✓	✓	High
Romano et al.^(35)^	✓	✓	✓	✓	?	✓	✓	✓	High
Seweryn et al.^(36)^	✓	✓	✓	✓	?	✓	✓	✓	High
Siracusano et al.^(37)^	✓	✓	✓	✓	?	✓	✓	✓	High
Sousa-Escandón et al.^(38)^	✓	✓	✓	✓	?	✓	✓	✓	High

### Studies excluded

The study by Balci et al.,^39^ evaluated a type of sling that did not meet the inclusion criteria. The other studies were excluded for not having reported the primary outcome of this review or having presented participants with less than 12 months of follow-up^40-48^(Table 8).

**Table 8:** Characteristics of the excluded studied

Balci et al.^(39)^	Type of sling different from inclusion criterion
Dalpiaz et al.^(40)^	No report of primary review outcome
Friedl et al.^(41)^	No report of primary review outcome
González et al.^(42)^	Follow up for less than 12 months
Hübner et al.^(43)^	No report of primary review outcome
Krause et al.^(44)^	No report of primary review outcome
Kretschmer et al.^(45)^	No report of primary review outcome
Miodrag et al.^(46)^	Follow up for less than 12 months
Jiménez Parra et al.^(47)^	Follow up for less than 12 months
Romano et al.^(48)^	Follow up for less than 12 months

### Effect of intervention

The RCT^21^ compared the Argus T^®^ (n=11) adjustable sling with the Advance^®^ non-adjustable sling (n=11). After a follow-up period of 18 months, the authors verified cure (0-1 absorbent pad in 24 hours) in 77.8% of patients who received Argus T^®^ and in 45.5% of the group that received the Advance^®^ implant; this difference was not significant. There were also no significant differences in the rates of complications between the two intervention groups.

Of the case series studies included, five^22,27,28,32,36^evaluated the ATOMS^®^ model, five^24-26,31,34^ Argus^®^, three^23,35,37^ Argus T^®^, three^30,33,38^ the Remeex^®^, and one,^29^ MRS^®^. The follow-up period varied from 12 months to 46 months. Heterogeneity was verified in the clinical characteristics of patients included as to gravity of PPI, radiation, and surgery for prior PPI.

**Figure 2:**
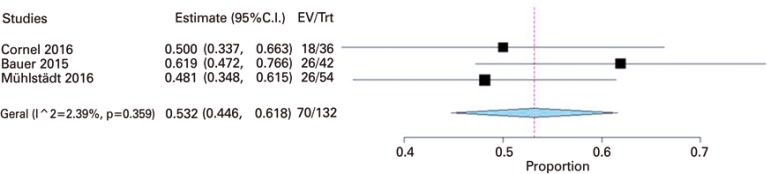
Meta-analysis of cure as 0 pad-test in 24 hours

The primary outcome, cure, defined as 24-hour pad test equal to zero, was reported by three studies,^23,26,32^ and the meta-analysis resulted in a cure rate of 53% (95%CI: 45%-62%; 132 participants). This analysis did not present with heterogeneity (I^2^=0%, p=0.36), and the fixed model of analysis was used (Figure 2).

**Figure 3:**
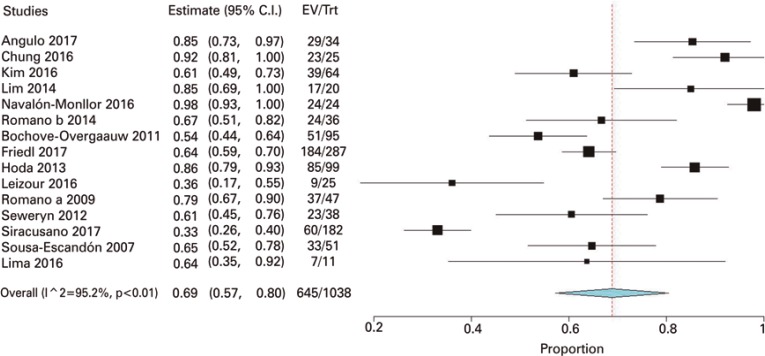
Meta-analysis of cure as 0-1 pads test in 24 hours

For the primary outcome, defined as cure meaning 0-1 pad in 24 hours, 15 studies were included. A meta-analysis demonstrated cure of 69% (95%CI: 57%-80%; 1,038 participants). This analysis presented with heterogeneity (I^2^=95.2%, p<0.01) and, thus, the random model was used (Figure 3).

The proportion of participants who required tension adjustments in the sling during follow-up was reported in nine studies,^22,24,30,31,33-35,37,38^varying from 19.4% to 60%. The mean readjustments necessary varied from 1.5 to 4.5 times.

**Figure 4:**
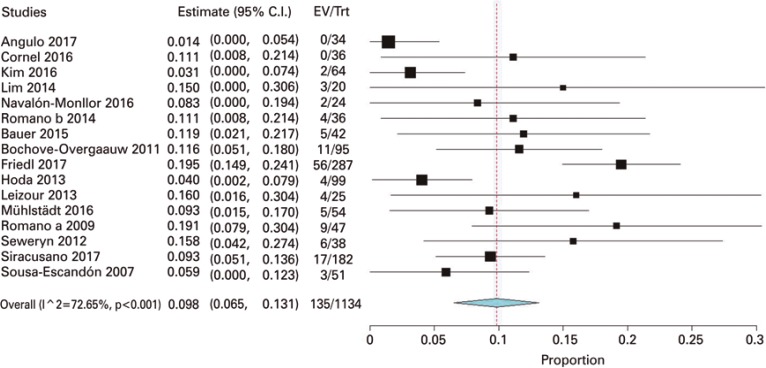
Meta-analysis of sling extrusion rate in the postoperative period

The rate of extrusion in the postoperative phase was reported in 16 studies, and meta-analysis resulted in 9.8% (95%CI: 6.5%-13.1%; 1,134 participants; I^2^=72.6%, random modelo) (Figure 4).

The most often reported complications were local postoperative pain of implantation, with a frequency of 1.7% to 30%, and local infection, with frequencies of 4% to 12%.

Table 9 displays a summary of results with the quality of evidence.

**Table 9:** Summary of results

Outcome	Participant (studies)	% (IC95%)	Quality of evidence	Comment
Pad-test 24 hours = 0	132 (3 studies)	53 (45-62)	Low	Descriptive studies
				Limited sample
Pad-teste 24 hours = 0-1	1,038 (13 studies)	69 (57-80)	Low	Descriptive studies
				Heterogeneity in analyses
Extrusion rate	1,134 (16 studies)	9.8 (6,5-13.1)	Low	Descriptive studies
				Heterogeneity in analyses

## DISCUSSION

The quality of evidence of effectiveness and safety of the adjustable slings in the treatment of PPI is low. Only one RCT with a high risk of bias was located and concluded the equivalence of effectiveness of adjustable and non-adjustable slings. All the other studies were case studies − most with a small sample size. Additionally, the studies made were heterogeneous in the groups studied, with patients of various levels of intensity of urinary incontinence and a history of radiation and prior operations. Only the meta-analysis for 0 pad in 24 hours demonstrated homogeneity with an effectiveness of 53%. For the outcome of 0 to 1 pad in 24 hours, the meta-analysis presented with heterogeneity and resulted in an effectiveness of 69%. The risk factors observed in the studies for unsuccessful surgery included prior pelvic irradiation, severity of PPI, and prior operations. The meta-analysis of sling extrusion rate during follow-up was 9.8%, and the most often reported complications were pain and local infection.

We believe that this systematic review was comprehensive, since we investigated the major databases of studies, including one relevant to our region (LILACS). There was no restriction as to language or date of publication. Nonetheless, the localization of studies, most of them descriptive, resulted in low quality evidence, and highlighted the need for research in the area.

The results observed in this review were similar to those noted in a systematic review with metanalysis performed by Chen et al.,^49^ which jointly analyzed all types of sling and found a cure rate of 60% (95%CI: 51%-67%).

Implantation of an artificial urinary sphincter has so far been considered the gold standard of surgical treatment for PPI, especially for cases of severe incontinence. A systematic review published by Van der Aa et al.,^50^ included case series studies with a minimum of follow-up of 2 years and noted effectiveness of 0 to 1 pad in 24 hours of 79% (95%CI: 60%-100%), with a rate of erosion and infection of 8.5%, mechanical failure of 6.2%, and need for reintervention of 26%. These results show that when this is the option to be considered, the adjustable slings display similar effectiveness and safety profiles, even when including patients with severe and irradiated cases of PPI.

A national study in the United States^51^ assessed 1,246 beneficiaries of Medicare between 2000 and 2011 diagnosed with PPI, and identified that the mean proportion of 35% of patients that received an artificial urinary sphincter implantation maintained stability during the decade, but the proportion of patients that received the sling increased drastically, from 14.8% to 51.4%. Another study^52^ evaluated the preference of the patient with PPI, and of 24 patients informed about the pros and cons of the artificial urinary sphincter implantation and of the sling, 22 (92%) chose the sling; of 63 patients who, due to their characteristics had a medical indication for the artificial sphincter implant, even so, 25% chose the sling. The rationale for the choice of the sling was the preference of avoiding handling of the mechanical equipment. Recent systematic reviews of surgical treatments for PPI concluded that the adjustable slings should be considered as a preferential option for patients with light to moderate incontinence, and in those who do not desire or cannot receive the implantation of the artificial urinary sphincter.^13,14^

Thus, when indicating surgical treatment for PPI, the patient should be informed about the current limited quality of evidence in the area, success rates, and complications of each option, and along with the physician, evaluate the best procedure to be adopted.

## CONCLUSION

Low-quality evidence indicates that the adjustable slings are effective for treatment of post-prostatectomy urinary incontinence, with a frequency of adverse events similar to those of the surgical option considered the gold standard (implantation of the artificial urinary sphincter). Further randomized comparative studies are warranted, with a standardized definition of severity of urinary incontinence, as well as methods of outcome measurements. Other studies should enable the analysis of a subgroup of patients, according to severity, irradiation, and prior surgeries, to indicate better the procedure according to the characteristics and desire of each patient.
