# Supplementary material for: Adjustable sling for the treatment of post-prostatectomy urinary incontinence: systematic review and meta-analysis
Source: Einstein (Sao Paulo). 2019 Sep 16;17(4):eRW4508. doi: 10.31744/einstein_journal/2019RW4508 (PMC6748345; doi:10.31744/einstein_journal/2019RW4508)
Supplement: Translation

# Translation: Implante de *sling* ajustável no tratamento da incontinência urinária pós-prostatectomia: revisão sistemática e meta-análise

**Authors:** Laercio Antonio da Silva, Rogério Simonetti, Edina Mariko Koga da Silva

**Affiliations:** 1 Brasil Hospital Israelita Albert Einstein, São Paulo, SP, Brasil.; 2 Brasil Universidade Federal de São Paulo, São Paulo, SP, Brasil.

**Keywords:** Slings *s*uburetrais, Incontinência urinária, Prostatectomia

## Abstract

A incontinência urinária pós-prostatectomia tem importante impacto negativo na qualidade de vida do portador. O tratamento cirúrgico inclui *slings* masculinos e, entre os diversos modelos, os *slings* ajustáveis. O objetivo deste estudo foi avaliar a efetividade e a segurança do *sling* ajustável no tratamento da incontinência urinária pós-prostatectomia. Trata-se de revisão sistemática de literatura. Foram pesquisadas as seguintes bases de dados eletrônicas até janeiro de 2018: PubMed^®^, Embase, CENTRAL e LILACS. As palavras-chaves utilizadas nas estratégias de busca foram: “*prostatectomy*” [Mesh], “*urinary incontinence*” [Mesh] e “*suburethral slings*” [Mesh]. Foram incluídos ensaios clínicos randomizados e estudos observacionais controlados ou não com seguimento maior que 12 meses. Apenas um estudo randomizado com alto risco de viés foi localizado e concluiu a equivalência de efetividade de *slings* ajustáveis e não ajustáveis. Todos os outros estudos foram série de casos com pacientes de variados níveis de intensidade da incontinência e histórico de radioterapia pélvica e cirurgias prévias. A metanálise para 0 pad em 24 horas demonstrou efetividade de 53%. Para o desfecho 0 a 1 pad em 24 horas, a metanálise resultou em efetividade de 69%. Os fatores de risco para insucesso da cirurgia incluem radiação prévia, gravidade da incontinência urinária pós-prostatectomia e cirurgias anteriores. A metanálise da taxa de extrusão foi de 9,8%, e os efeitos adversos mais relatados foram dor e infecção local. Evidências de baixa qualidade indicam que os *slings* ajustáveis são efetivos para o tratamento da incontinência urinária pós-prostatectomia, com frequência de eventos adversos semelhantes à opção cirúrgica considerada padrão-ouro (o implante de esfíncter urinário artificial).

## INTRODUÇÃO

A incontinência urinária como sequela de cirurgias de próstata por doença benigna ou maligna é bem conhecida, sendo um desfecho frequentemente temido e com impacto negativo significante na qualidade de vida do portador.^(1)^ A maioria dos pacientes com incontinência urinária pós-prostatectomia (IUPP) tem deficiência intrínseca do esfíncter isolada ou associada com disfunção do detrusor^(2)^ e apresenta incontinência urinária por estresse, que é a queixa de perda involuntária de urina aos esforços.^(3)^

As taxas de incontinência urinária após a cirurgia por doença benigna de próstata mostraram-se semelhantes nas várias modalidades de tratamento cirúrgico, tendendo a ser ligeiramente maiores após prostatectomia aberta − de 0% a 8,4%.^(4)^ A incidência de incontinência urinária após prostatectomia radical é fonte de controvérsia, porque as taxas apresentadas nos diversos estudos têm variado muito, de 0,8 a 87%.^(5-11)^ Essa grande variação é provavelmente devida à falta de definição padronizada de incontinência urinária, tempo de evolução pós-cirúrgica, método de diagnóstico e características da população estudada.

O tratamento cirúrgico da IUPP deve ser indicado somente após 6 a 12 meses da cirurgia prostática. Durante esse período terapias conservadoras como treinamento da musculatura do assoalho pélvico, intervenções no estilo de vida e *biofeedback* devem ser considerados.^(12)^ As opções de tratamento cirúrgico incluem injeção de substâncias que causam aumento volumétrico, ocluindo porções da uretra, *slings* masculinos, implante de balões infláveis que ocluem parcialmente a uretra e implante de aparelho esfincteriano artificial.

O esfíncter urinário artificial tem demonstrado resultados favoráveis em longo prazo, sendo considerado padrão-ouro para a IUPP até o momento. No entanto, essa opção apresenta taxas bem estabelecidas de complicações cirúrgicas, incluindo erosão uretral (6%), infecções (5%), falhas mecânicas (6%) e necessidade de revisão de 21% em 5 anos e de 50% em 10 anos.^(13)^ Assim, nos últimos anos, o uso de *slings* masculinos tem aumentado, por oferecer opção de intervenção mais simples, menos invasiva e sem a necessidade de manipulação de dispositivo mecânico pelo paciente.

Vários modelos de *slings* masculinos foram lançados na última década e, apesar dos diferentes mecanismos de ação, o objetivo primário é a compressão com tensão adequada do bulbo uretral, com manutenção da tensão para prevenção de vazamentos e equilíbrio entre a tensão e a contractilidade do detrusor, para evitar a retenção urinária.^(13)^ Atualmente, os modelos disponíveis de *slings* masculinos podem ser divididos como suburetral transobturador e os mais recentes retropúbicos ajustáveis.

Os *slings* retropúbicos ajustáveis são geralmente inseridos em posição suburetral, sob o músculo bulboesponjoso, pressionando principalmente a uretra bulbar e, em menor extensão, a uretra membranosa. No seguimento pós-operatório, se a tensão não for suficiente para alcançar a continência, ela pode ser reajustada por um procedimento minimamente invasivo específico de cada modelo.^(14)^ Os *slings* ajustáveis disponíveis incluem Argus^®^ (Promedon, Córdoba, Argentina), ArgusT^®^ (Promedon, Córdoba, Argentina), ReMeex^®^ (Neomedic, Barcelona, Espanha) e ATOMS^®^ (AMI, Feldkirch, Áustria).

Vasta literatura sobre esses *slings* tem sido publicada recentemente e, segundo os relatos, eles seriam efetivos, mesmo em casos de IUPP grave e em pacientes que receberam radiação prévia. No entanto, nenhuma revisão sistemática específica para esses modelos foi publicada até o momento.

## OBJETIVO

Avaliar a efetividade e a segurança dos *slings* masculinos ajustáveis no tratamento da incontinência urinária pós-prostatectomia.

## MÉTODOS

Esta revisão sistemática com meta-análise foi conduzida de acordo com o protocolo PRISMA^(15)^ e registrada no *International Prospective Register of Systematic Reviews* (PROSPERO) com o identificador CRD42017082431.

### Tipos de estudo

Foram incluídos ensaios clínicos randomizados e estudos observacionais, controlados ou não, que avaliaram a cirurgia de implantação de *sling* ajustável para o tratamento de IUPP. Apenas estudos com mais de 12 meses de seguimento foram selecionados.

### Tipo de participantes

Foram considerados elegíveis estudos com adultos que apresentam IUPP há mais de 6 meses, sem melhora com tratamento conservador.

### Tipo de intervenção

Foram incluídos estudos que avaliaram a cirurgia de implantação de *sling* ajustável, comparada ou não a qualquer outro tipo de cirurgia para o tratamento da IUPP.

### Tipos de medidas de resultado

O desfecho primário foi cura avaliada pelo instrumento padronizado pad-teste em 24 horas.^(16)^ Os desfechos secundários incluíram eventos adversos e necessidades de ajustes no acompanhamento.

### Pesquisa para identificação de estudos

Foram realizadas pesquisas nos bancos de dados até janeiro de 2018: PubMed^®^, EMBASE, *Cochrane Central Register of Controlled Trials* (CENTRAL) e Literatura Latino-Americana e do Caribe em Ciências da Saúde (LILACS) (Tabelas 1 a 4 [Table t2002] [Table t3002] [Table t4002]). Foi realizada busca de estudos em andamento no Site ClinicalTrials (https://clinicaltrials.gov/). Não houve restrição de língua e nem de data de publicação. As listas de referências de estudos relevantes foram verificadas para possível localização de estudos.

**Tabela 1:** Termos de busca utilizados em todos os bancos de dados

#1 − *(Surgery OR operative therapy OR operative procedures OR invasive procedures OR operations OR peroperative procedures OR perioperative procedures OR preoperative procedures OR Intraoperative procedures OR Operative Surgical Procedure OR Operative Surgical Procedures OR Operative Procedures OR Operative Procedure)*
*#2 – (post prostatectomy OR post-prostatectomy OR Prostatectomy OR Prostatectomies OR Suprapubic Prostatectomies OR Suprapubic Prostatectomy OR Retropubic Prostatectomies OR Retropubic Prostatectomy)*
#3 − *(Urinary Urge Incontinence OR Urge Incontinence OR Urinary Reflex Incontinence OR Urinary Stress Incontinence)*
*#4 − (suburethral Slings OR Suburethral Sling OR Transobturator Tape OR Transobturator Tapes OR Transobturator Suburethral Tape OR Trans-Obturator Tape)*
*#5 #1 AND #2 AND #3 AND #4*

**Tabela 2 t2002:** Estratégia de busca MEDLINE via PubMed®

*#1 randomised controlled trial [pt]*
*#2 controlled clinical trial [pt]*
*#3 randomized [tiab]*
*#4 placebo [tiab]*
*#5 drug therapy [sh]*
*#6 randomly [tiab]*
*#7 trial [tiab]*
*#8 groups [tiab]*
*#9 #1 OR #2 OR #3 OR #4 OR #5 OR #6 OR #7 OR #8*
*#10 animals [mh] NOT humans [mh]*
*#11 #9 NOT # 10*

**Tabela 3 t3002:** Estratégia de busca Literatura Latino-Americana e do Caribe em Ciências da Saúde (LILACS) via Centro Latino-Americano e do Caribe de Informação em Ciências da Saúde (BIREME)

*(Pt randomized controlled trial OR Pt controlled clinical trial OR Mh randomized controlled trials OR Mh random allocation OR Mh double-blind method OR Mh single-blind method) AND NOT (Ct animal AND NOT (Ct human and Ct animal)) OR (Pt clinical trial OR Ex E05.318.760.535$ OR (Tw clin$ AND (Tw trial$ OR Tw ensa$ OR Tw estud$ OR Tw experim$ OR Tw investiga$)) OR ((Tw singl$ OR Tw simple$ OR Tw doubl$ OR Tw doble$ OR Tw duplo$ OR Tw trebl$ OR Tw trip$) AND (Tw blind$ OR Tw cego$ OR Tw ciego$ OR TW Mask$ or Tw mascar$ )) OR Mhplacebos PR Twplacebo$ OR (Tw random$ OR Twrandon$ OR Tw casual$ OR Tw acaso$ OR Tw azar OR Tw aleator$) OR Mh research design) AND NOT (Ct human and Ct animal)) OR (Ct comparative study OR Ex E05.337$ OR Mh follow-up studies OR Mh prospective studies OR Tw control$ OR Tw prospectiv$ OR Tw volunt$ OR Tw volunteer$) AND NOT (Ct animal AND NOT (human and Ct animal)))*

**Tabela 4 t4002:** Estratégia de busca EMBASE via Ovid®

*01 random$*
*02 factorial$*
*03 crossover$*
*04 cross over$*
*05 cross-over$*
*06 placebo$*
*07 double$ adj blind$*
*08 singl$ adj blind$*
*09 assign$*
*10 allocate$*
*11 volunteer$*
*12 cross-over procedure*
*13 double-blind procedure*
*14 randomized controlled trial*
*15 single-blind procedure*

### Seleção de estudos

Dois autores avaliaram independentemente os estudos identificados pela pesquisa bibliográfica para a elegibilidade. Se ocorresse alguma incerteza sobre a elegibilidade dos estudos com base no título e no resumo, o trabalho completo era obtido e examinado pelos dois revisores. Um terceiro autor foi consultado em caso de desacordo, até que um consenso fosse alcançado. Todos os estudos considerados elegíveis foram obtidos na íntegra e analisados.

### Avaliação metodológica dos estudos incluídos

Dois autores avaliaram os estudos incluídos de forma independente para a qualidade metodológica. Para avaliação da qualidade metodológica de ensaios clínicos randomizados, foi utilizando a ferramenta da Colaboração Cochrane^(17)^ e, para estudos observacionais, foi utilizado o instrumento para avaliação crítica de estudos do tipo série de casos de Chan et al.^(18)^

### Síntese e análise de dados

Foram calculadas medidas de frequência absoluta e relativa com intervalos de confiança de 95% (IC95%). Para os resultados de variáveis contínuas as tendências centrais e variação com IC95% foram calculadas. A unidade de análise baseou-se no paciente individual. Para realização de meta-análise dos dados extraídos, foi utilizado o software *OpenMetaAnalyst.*^(19)^

### Avaliação de heterogeneidade

Para quantificar as inconsistências entre as estimativas somadas, utilizou-se o teste de I^2^=[(Q-gl)/Q] × 100%, onde “Q” é a estatística χ^2^ e “gl” são seus graus de liberdade. Isto ilustra a percentagem da variabilidade nas estimativas de efeito resultante da heterogeneidade.^(20)^ Foi utilizado o modelo fixo na ausência de heterogeneidade substancial (I^2^<50%), e o modelo randômico quando houve heterogeneidade (I^2^≥50%).

## RESULTADOS

A estratégia de busca recuperou 312 registros: PubMed^®^ com 177 referências; EMBASE, 113 referências; CENTRAL, 11 referências; e LILACS, 11 referências. Foram analisadas também as referências bibliográficas dos artigos relevantes para estudos potencialmente elegíveis e não se localizaram referências adicionais. Não foi localizado estudo em andamento. Após o exame dos títulos e resumos, eliminando as duplicatas dessas referências, 28 artigos foram selecionados para análise na íntegra. Foram excluídos 10 artigos por não preencherem os critérios de inclusão, e 18 estudos foram incluídos nessa revisão sistemática (Figura 1).

**Figura 1:** Diagrama do fluxo do estudo

### Desenho dos estudos incluídos

Apenas um ensaio clínico randomizado (ECR)^(21)^ com amostra limitada foi identificado na busca. Os outros estudos incluídos^(22-38)^ eram séries de casos prospectivos e retrospectivos. As características dos estudos incluídos estão descritas na tabela 5.

**Tabela 5:** Características dos estudos incluídos

Estudos	Tipo de *sling*	Desenho	Amostra	Gravidade da IUPP	Tempo de seguimento (meses)	Definição de cura	Taxa de cura (%)	Ajustes	Taxa de extrusão (%)
Lima et al.^(21)^	ArgusT^®^*versus* Advance^®^	Ensaio clínico randomizado	22	-	18	0-1 pad	ArgusT^®^: 77,8 Advance^®^: 45,5	Em 27,3%	-
Angulo et al.^(22)^	ATOM^®^	Prospectivo	34	Leve: 5,9%	18,5	0-1 pad	85,3	1,0 vez	0
				Moderada: 17,6%					
				Grave: 76,5%					
Bauer et al.^(23)^	ArgusT^®^	Prospectivo	42	Média: 7,1 pad/dia	28,8	0 pad	61,9	1,7 vez	4,8
Bochove-Overgaauw et al.^(24)^	Argus^®^	Retrospectivo	95	Leve: 13,7%	27	0-1 pad	54	1,5 vez	11,5
				Moderada: 48,2%					
				Grave: 43,1%					
Chung et al.^(25)^	Argus^®^	Prospectivo	25	-	36,2	0-1 pad	92	-	-
Cornel^(26)^	Argus^®^	Prospectivo	36	-	12	0 pad	50	-	11,1
Friedl et al.^(27)^	ATOMS^®^	Retrospectivo	287	Leve: 11%	31	0-1 pad	64	3,0 vezes	20
				Moderada: 67%					
				Grave: 22%					
Hoda et al.^(28)^	ATOMS^®^	Prospectivo	99	Leve: 12%	17,8	0-1 pad	85,5	3,8 vezes	4
				Moderada: 39%					
				Grave: 49%					
Kim et al.^(29)^	MRS^®^	Prospectivo	64	Leve: 42,2%	46	0-1 pad	60,9	1,9 vezes	3,1
				Moderada: 43,8%					
				Grave: 14%					
Leizour et al.^(30)^	Remeex^®^	Prospectivo	25	Leve: 60%	31	0-1 pad	36	Em 60%	16
				Moderada: 20%					
				Grave: 20%					
Lim et al.^(31)^	Argus^®^	Prospectivo	20	Moderada: 100%	24,7	0-1 pad	85	Em 45%	15
Mühlstädt et al.^(32)^	ATOMS^®^	Retrospectivo	54	Leve: 1,9%	27,5	0 pad	48	4,5 vezes	9,3
				Moderada: 29,6%					
				Grave: 68,5%					
Navalón-Monllor et al.^(33)^	Remeex^®^	Prospectivo	24	Grave: 100%	40,7	0-1 pad	100	2,4 vezes	8
Romano et al.^(34)^	Argus^®^	Prospectivo	47	-	45	0-1 pad	78,7	Em 19,4%	19,1
Romano et al.^(35)^	ArgusT^®^	Prospectivo	36	Leve e moderada: 22%	45	0 pad	66	Em 19,4%	10,4
				Grave: 78%					
Seweryn et al.^(36)^	ATOMS^®^	Prospectivo	38	Leve: 7,9%	17	0-1 pad	60,5	3,9 vezes	15,8
				Moderada: 34,2%					
				Grave: 57,9%					
Siracusano et al.^(37)^	ArgusT^®^	Prospectivo	182	Leve: 11,6%	22	0-1 pad	33	Em 30%	9,3
				Moderada: 52,7%					
				Grave: 35,8%					
Sousa-Escandón et al.^(38)^	Remeex^®^	Prospectivo	51	-	32	0-1 pad	64,7	Em 33%	5,9

IUPP: incontinência urinária pós-prostatectomia.

### Participantes

Os estudos incluídos totalizaram 1.170 participantes, com idades que variaram entre 46 a 89 anos, com média aproximada de 70 anos de idade. Doze estudos incluíram pacientes que receberam radioterapia prévia, em uma proporção de 5% a 44,7%, e oito incluíram pacientes com cirurgia para IUPP prévia, em proporção de 11,8% a 36%. Doze estudos incluíram pacientes com IUPP grave, em proporção de 7,8% a 76,5%.

### Qualidade metodológica dos estudos incluídos

O único ECR^(21)^ apresentava baixa qualidade metodológica, devido à alocação incerta, falta de cegamento e amostra limitada (Tabela 6).

**Tabela 6:** Avaliação da qualidade do ensaio clínico randomizado(21)

Domínio	Julgamento	Descrição
Randomização adequada?	Sim	Tabela números randômicos
Ocultação de alocação?	Incerto	Não há informação
Cego?	Não	Estudo aberto
Dados de desfechos incompletos?	Não	Não relata perdas
Livre de desfecho seletivo	Sim	Desfechos relevantes de relatados em resultados
Livre de outros vieses	Não	Sem cálculo de tamanho de amostra Amostra limitada
Risco de viés	Alto	

Os 17 estudos do tipo série de casos apresentaram qualidade moderada à alta (Tabela 7).

**Tabela 7:** Avaliação da qualidade dos estudos incluídos

Estudo	Objetivo claro	Método adequado	Critérios de inclusão e exclusão	Período de recrutamento	Pacientes consecutivos	Desfechos apropriados	Prospectivo	Sem perdas importantes	Qualidade final
Angulo et al.^(22)^	✓	✓	✓	✓	✓	✓	✓	✓	Alta
Bauer et al.^(23)^	✓	✓	✓	✓	✓	✓	✓	✓	Alta
Bochove-Overgaauw et al.^(24)^	✓	✓	✓	✓	✓	✓	✓	✓	Alta
Chung et al.^(25)^	✓	✓	✓	✓	✓	✓	✓	✓	Alta
Cornel^(26)^	✓	✓	✓	✓	✓	✓	✓	✓	Alta
Friedl et al.^(27)^	✓	✓	✓	✓	?	✓	✓	✓	Moderada
Hoda et al.^(28)^	✓	✓	✓	✓	?	✓	✓	✓	Alta
Kim et al.^(29)^	✓	✓	✓	✓	✓	✓	✓	✓	Alta
Leizour et al.^(30)^	✓	✓	✓	✓	?	✓	✓	✓	Alta
Lim et al.^(31)^	✓	✓	✓	✓	?	✓	✓	✓	Alta
Mühlstädt et al.^(32)^	✓	✓	✓	✓	?	✓	✓	✓	Alta
Navalón-Monllor et al.^(33)^	✓	✓	✓	✓	?	✓	✓	✓	Moderada
Romano et al.^(34)^	✓	✓	✓	✓	?	✓	✓	✓	Alta
Romano et al.^(35)^	✓	✓	✓	✓	?	✓	✓	✓	Alta
Seweryn et al.^(36)^	✓	✓	✓	✓	?	✓	✓	✓	Alta
Siracusano et al.^(37)^	✓	✓	✓	✓	?	✓	✓	✓	Alta
Sousa-Escandón et al.^(38)^	✓	✓	✓	✓	?	✓	✓	✓	Alta

### Estudos excluídos

O estudo de Balci et al.,^(39)^ avaliou um tipo de *sling* que não atende aos critérios de inclusão. Os demais estudos foram excluídos por não reportarem o desfecho primário dessa revisão ou apresentarem participantes com menos de 12 meses de seguimento^(40-48)^ (Tabela 8).

**Tabela 8:** Características dos estudos excluídos

Balci et al.^(39)^	Tipo de sling diferente do critério de inclusão
Dalpiaz et al.^(40)^	Não reporta desfecho primário da revisão
Friedl et al.^(41)^	Não reporta desfecho primário da revisão
González et al.^(42)^	Seguimento inferior a 12 meses
Hübner et al.^(43)^	Não reporta desfecho primário da revisão
Krause et al.^(44)^	Não reporta desfecho primário da revisão
Kretschmer et al.^(45)^	Não reporta desfecho primário da revisão
Miodrag et al.^(46)^	Seguimento inferior a 12 meses
Jiménez Parra et al.^(47)^	Seguimento inferior a 12 meses
Romano et al.^(48)^	Seguimento inferior a 12 meses

### Efeito da intervenção

O ECR^(21)^ comparou o *sling* ajustável ArgusT^®^ (n=11) com o não ajustável Advance^®^ (n=11). Após um período de acompanhamento de 18 meses, os autores verificaram cura (0-1 pad absorvente em 24 horas) em 77,8% dos pacientes que receberam Argus T^®^ e de 45,5% no grupo que recebeu o implante de Advance^®^, sendo essa diferença não significante. Não existiram também diferenças significantes nas taxas de complicações entre os dois grupos de intervenção.

Dos estudos do tipo série de casos incluídos, cinco^(22,27,28,32,36)^ avaliaram o modelo ATOMS^®^, cinco^(24-26,31,34)^ o Argus^®^, três^(23,35,37)^ o ArgusT^®^, três^(30,33,38)^ o Remeex^®^ e um^(29)^ MRS^®^. O período de acompanhamento variou de 12 meses a 46 meses. Foi verificada heterogeneidade nas características clínicas dos pacientes incluídos quanto à gravidade da IUPP, radiação e cirurgia para IUPP anterior.

O desfecho primário cura, definido como pad-teste em 24 horas igual a zero, foi relatado por três estudos,^(23,26,32)^ e a meta-análise resultou em taxa de cura de 53% (IC95%: 45%-62%; 132 participantes). Essa análise não apresentou heterogeneidade (I^2^=0%, p=0,36), e foi utilizado o modelo fixo de análise (Figura 2).

**Figura 2:** Metanálise de cura como 0 pad em 24 horas

Para o desfecho primário definido para cura como 0-1 pad em 24 horas, foram incluídos 15 estudos. A meta-análise demonstrou taxa de cura de 69% (IC95%: 57%-80%; 1.038 participantes). Essa análise apresentou heterogeneidade (I^2^=95,2%, p<0,01) e, assim, o modelo randômico foi empregado (Figura 3).

**Figura 3:** Metanálise de cura como 0-1 pads em 24 horas

A proporção de participantes que necessitaram de ajustes de tensão do *sling* no seguimento foi relatada em nove estudos,^(22,24,30,31,33-35,37,38)^ variando de 19,4% a 60%. A média de números de reajustes necessários variou de 1,5 a 4,5 vezes.

A taxa de extrusão no pós-operatório foi relatada em 16 estudos, e a meta-análise resultou em 9,8% (IC95%: 6,5%-13,1%; 1.134 participantes; I^2^=72,6%, modelo randômico) (Figura 4).

**Figura 4:** Metanálise da taxa de extrusão do *sling* no pós-operatório

As complicações mais relatadas foram dor local de implantação no período pós-operatório, com frequência de 1,7% a 30%, e infecção local, com frequências de 4% a 12%.

Na tabela 9, apresentamos um sumário dos resultados com a qualidade da evidência.

**Tabela 9:** Sumário dos resultados

Desfecho	Participante (estudos)	% (IC95%)	Qualidade da evidência	Comentário
Pad-teste 24 horas = 0	132 (3 estudos)	53 (45-62)	Baixa	Estudos descritivos
				Amostra limitada
Pad-teste 24 horas = 0-1	1.038 (13 estudos)	69 (57-80)	Baixa	Estudos descritivos
				Heterogeneidade nas análises
Taxa de extrusão	1.134 (16 estudos)	9,8 (6,5-13,1)	Baixa	Estudos descritivos
				Heterogeneidade nas análises

## DISCUSSÃO

A qualidade da evidência de efetividade e segurança dos *slings* ajustáveis no tratamento da IUPP é baixa. Apenas um ECR com alto risco de viés foi localizado e concluiu a equivalência de efetividade de *slings* ajustáveis e não ajustáveis. Todos os outros estudos eram série de casos − a maioria com pequeno tamanho amostral. Além disso, os estudos foram heterogêneos nos grupos estudados, com pacientes de variados níveis de intensidade de incontinência urinária e histórico de radiação e cirurgias prévias. Apenas a meta-análise para 0 pad em 24 horas demonstrou homogeneidade com efetividade de 53%. Para o desfecho 0 a 1 pad em 24 horas, a meta-análise apresentou heterogeneidade e resultou em efetividade de 69%. Os fatores de risco observados nos estudos para insucesso da cirurgia incluíram radiação pélvica prévia, gravidade da IUPP e cirurgias anteriores. A meta-análise da taxa de extrusão do *sling* no seguimento foi de 9,8%, e as complicações mais relatadas foram dor e infecção local.

Acreditamos que essa revisão sistemática foi abrangente, pois foram pesquisados os principais banco de dados de estudos, incluindo um relevante para o nosso meio (LILACS). Não houve restrição de idioma e nem de data da publicação. No entanto, a localização de estudos em sua maioria descritivos resulta em evidência de baixa qualidade e ressalta a necessidade de pesquisas na área.

Os resultados observados nesta revisão foram semelhantes aos observados em revisão sistemática com metanálise realizada por Chen et al.,^(49)^ que analisaram em conjunto todos os tipos de *sling* e observaram taxa de cura de 60% (IC95%: 51%-67%).

O implante de esfíncter urinário artificial é, até o momento, considerado o padrão-ouro de tratamento cirúrgico de IUPP, sobretudo para os casos de incontinência grave. Revisão sistemática publicada por Van der Aa et al.,^(50)^ incluiu estudos do tipo séries de casos com um mínimo de 2 anos de acompanhamento e observou efetividade para 0 a 1 pad em 24 horas de 79% (IC95%: 60%-100%), com taxa de erosão e infecção de 8,5%, falha mecânica em 6,2% e necessidade de reintervenção de 26%. Esses resultados evidenciam que os *slings* ajustáveis apresentam resultados de efetividade e perfil de segurança semelhantes, mesmo incluindo pacientes com IUPP graves e irradiados, sendo opção a ser considerada.

Estudo nacional dos Estados Unidos^(51)^ avaliou 1.246 beneficiários do Medicare entre 2000 e 2011 com diagnóstico de IUPP, e verificou que a proporção média de 35% de pacientes que receberam implante de esfíncter urinário artificial se manteve estável na década, mas a proporção de pacientes que receberam *sling* aumentou drasticamente, de 14,8% para 51,4%. Outro estudo^(52)^ avaliou a preferência do paciente com IUPP, e de 24 pacientes informados sobre os prós e os contra do implante de esfíncter urinário artificial e do *sling*, 22 (92%) escolheram o *sling*; de 63 pacientes que, devido a suas características tiveram indicação médica para o implante de esfíncter urinário artificial, 25% mesmo assim escolheram o *sling*. A justificativa mais referida para a escolha de *sling* foi a preferência de evitar o manuseio de equipamento mecânico. Recentes revisões sistemáticas dos tratamentos cirúrgicos para a IUPP têm concluído que os *slings* ajustáveis devem ser considerados como opção preferencial para pacientes com incontinência leve à moderada e naqueles que não desejam ou não têm condições de receber implante de esfíncter urinário artificial.^(13,14)^

Assim, ao se indicar o tratamento cirúrgico para a IUPP, o paciente deve ser informado da atual limitada qualidade da evidência na área, das taxas de sucesso e de complicações de cada opção e, juntamente do médico, avaliar o melhor procedimento a ser realizado.

## CONCLUSÃO

Evidências de baixa qualidade indicam que os *slings* ajustáveis são efetivos para o tratamento da incontinência urinária pós-prostatectomia, com frequência de eventos adversos semelhantes aos da opção cirúrgica considerada padrão-ouro (o implante de esfíncter urinário artificial). Existe a necessidade de estudos comparativos randomizados, com definição padronizada de gravidade de incontinência urinária, assim como dos métodos de medidas de desfechos. Os estudos devem possibilitar a análise de subgrupo de pacientes, segundo gravidade, irradiação e cirurgias anteriores, para melhor indicação de procedimento conforme a característica e a vontade de cada paciente.
